# Editorial

**Published:** 1866-11

**Authors:** 


### Editorial.

DEATH OF PROF. D. BRAINARD.—With feelings of deep sadness we record the death of one of the most prominent surgeons in our country. DANIEL BRAINARD, so long at the head of the department of surgery in the North-west, was attacked with epidemic cholera on Tuesday night, and died on Wednesday afternoon, the 10th inst. Although only 53 years of age, a period which should be the vigor of active manhood, it was generally understood that Dr BRAINARD'S health had not been good for one or two years past.

At the commencement of his professional career, he took special pains to qualify himself for the practice of surgery, and almost immediately became identified with the establishment of Rush Medical College in this city; the Surgical Chair in which he filled with marked ability until the day before his death. He was never popular as a general practitioner of medicine, and during the last *ten* years of his life his professional practice was restricted almost exclusively to surgical cases. He was not a showy or brilliant operator, but a deliberate, accurate, and successful surgeon. It was in the lecture-room, however, in his capacity as teacher, that he achieved the highest reputation. Possessed of a mind well stored by reading and observation, and thoroughly disciplined by reflection, his didactic instruction was clear, concise, and impressive; giving him an enviable popularity as a lecturer. In his unofficial capacity, as a member of the profession, Dr. BRAINARD was not noted for genial affability, scrupulous regard for ethical rules, or cordial coöperation in the support of medical organizations. He was not only studious in everything relating to his favorite department, but he often entered boldly upon original investigations and experiments concerning such topics as strongly attracted his attention. Though he wrote no complete work upon any department of medical science, yet the results of his experiments in the treatment of ununited fractures; on the effects of iodine on the poison of serpents; and on the effects of lactate of iron injected into the veins, in the treatment of the cancerous diathesis, will perpetuate his name on all the future annals of our professional literature. Although the strictly professional income of Dr. BRAINARD was never large, yet, by some successful real estate operations, he had secured to himself and his family an ample fortune.

The action taken by the profession of our city, and also by that of Springfield, in honor of the deceased, will be found on the following pages of this number of the EXAMINER.

---

THE DEATH OF DANIEL BRAINARD, M.D.—MEETING OF THE MEDICAL PROFESSION.—A meeting of the members of the medical profession was held on the afternoon of Thursday, Oct. 11th, at the Common Council room, to take suitable action in regard to the sudden death of DANIEL BRAINARD, M.D., who was recently stricken down in the maturity of his powers and the zenith of his honors, by the cholera. The meeting was organized by the appointment of Dr. Duck, as Chairman, and Dr. Charles G. Smith, as Secretary.

On motion, the Chair appointed a committee of five to draft resolutions expressive of the regret of the profession at the demise, and evidencing their sorrow and grief at the dread visitation which had thus inopportunely carried away an ornament to the profession.

The following gentlemen were designated as such committee: Drs. McVicker, Johnson, Trimble, Smith, and Byford, and after a brief absence they presented the following resolutions, which were unanimously adopted:—

*Resolved*, That in the dispensation of Divine Providence, who has removed from our midst our deceased friend and brother,

Daniel Brainard, we recognize the hand of Him who doeth all things well, and we bow submissively to His will.

*Resolved,* That our deceased brother, by his natural powers of mind, by his capacity as a teacher, by his industry, by his untiring zeal and assiduity in the profession of which he was a distinguished member and ornament, has acquired a position of character and usefulness, recognized not only by his colleagues at home, but by the profession throughout the world.

*Resolved,* That, in his example, we read the great lesson of encouragement in the path of duty and honor, and, while humbly seeking to imitate it, we hold it up to the consideration of members of his own and other professions elsewhere, and that in his death the profession of our city and the North-west has suffered an irreparable loss.

After the adoption of the resolutions, Dr. BROCK McVICKAR paid the following feeling tribute to the memory of the deceased:

EULOGY BY DR. BROCK MCVICKAR.

The occurrence which has called us together this afternoon, is recited in brief aud emphatic words in the daily papers of our city:—Died, in this city, Oct. 10, of cholera, Daniel Brainard, M.D., aged 53 years.

When we were last assembled in this room, gentlemen, it was to record our respect for a departed brother who had been called away in the spring-time of professional life, with the future all bright and promising before him. To-day, we come to pay the same sad office to another, one older in years and full of honors, whose ambition and aspirations of the future had become to him fruition in the present, and who stood, with well-earned laurels, at the head of a profession, which he dignified and crowned.

Daniel Brainard was no ordinary man; he was possessed of high and sterling ability, quickened and developed by culture and study; of untiring industry, of unremitting perseverance and unflagging zeal in the pursuit of knowledge. He was called cold and reticent at times, but what seemed coldness to many was but the absorption of a strong and earnest nature, pushing forward to the attainment of the high mark he had proposed to himself. He had an abiding hatred for pretenders and shams, and looked with little favor upon those who followed their profession without seeking to rise above its daily routine of duty; when once satisfied of a man's integrity, and earnestness, and intelligent devotion to his profession, he was his friend.

With Dr. Brainard, with but a slight interruption, my relations, personal and professional, have always been of the most agreeable character; and, at the time of his death, they were particularly so; and I shall never forget how, during my last interview with him, but a few days since, I was impressed with respect and regard for him, as a gentleman courtly in his manners, polished by foreign travel and culture, the peer of the highest in the land.

His career, gentlemen, may serve to illumine the pathway of the student, to quicken him to higher and better efforts, and give him assurance of success. Of Brainard's success, much feeling has been manifested by smaller minds, many times falling under my own notice. He was restless sometimes under this jealousy and its resulting injustice, and that restlessness toned and tempered his feelings; but when a man has attained the measure of success which fell to his lot, and which he earned so faithfully, he can afford to smile instead of being annoyed, and look down, as he did in his later years, with the calm consciousness of superiority, grafted by his own hand and his own exertions, or powers and faculties given him by God. But he is gone from us, and the places which knew him once shall know him no more again forever. Peaceful be his rest, green be the turf which grows above him, and bright be the place which he shall hold in the memory of the friends he has left behind him.

Drs. Davis, Johnson, and Paoli followed Dr. McVickar, and spoke feelingly and affectionately of the manifold virtues of the deceased, his exalted professional ability, and the endearing reminiscences which would ever follow his memory in their hearts.

Dr. Blaney presented a request from Dr. Powell, the nephew and only surviving relative of deceased in the city, soliciting the meeting to designate six members of the profession to officiate as pall-bearers at the funeral.

The Chair, in consonance with the sense of the meeting, appointed the following gentlemen:—Drs. Hitchcock, Duck, Eldredge, Johnson, Paoli, and Hamill.

Dr. Johnson submitted to the meeting a proposition relative to taking a cast of the features of deceased, previous to interment, and their lasting perpetuation either in statuette or bust models. Several gentlemen expressed their approbation of the movement, and, in pursuance thereof, a committee, consisting of Drs. Hamill, Johnson, Blaney, Davis, and Miller, was appointed to carry the design into effect.

The meeting then adjourned.

---

Meeting of the Sangamon County Medical Society.—Resolutions of Respect.—A special meeting of the Sangamon County Medical Society was held in the city of Springfield, October 13th, at 3 P.M. The President being absent, Dr. Wright, of Chatham, Vice-President, took the Chair.

On motion of Dr. Wardner, the Chairman appointed a Committee of three to draft suitable resolutions regarding the death of Dr. Daniel Brainard, of Chicago. The Committee consisted of Drs. Wardner, Griffith, and Bailache.

On motion of Dr. Townsend, Dr. Wright was added to that Committee.

The Committee reported back the following preamble and resolutions, which were unanimously adopted:—

*Whereas*, It has pleased an All-Wise Providence to remove by death, Daniel Brainard, M.D., late President and Professor in Rush Medical College, whose ability and professional attainment gave him high rank in medical science, not only among friends and acquaintances, but among all devotees of the science he so much honored, therefore,

*Resolved*, That in the death of Dr. Brainard, the profession is bereft of an able teacher, science of an ardent student, and the community in which he lived of a valuable citizen.

*Resolved*, That we tender to the faculty of Rush Medical College our condolence for the loss of its President, and one of its most honored teachers.

*Resolved*, That we tender to the afflicted family our sympathies in this, their hour of sad bereavement.

*Resolved*, That the Secretary furnish a copy of these resolutions to the family of the deceased, and the faculty of Rush Medical College. Also, a copy to each of the papers of this place, and to the *Chicago Medical Examiner* and *Chicago Medical Journal*, for publication.

N. WRIGHT, *Chairman*.

A. L. Converse, *Secretary*.

---

### REPORT OF THE COMMITTEE ON THE SANITARY CONDITION OF THE CITY, AND THE PREVAALENCE OF CHOLERA, DURING THE MONTHS OF AUGUST, SEPTEMBER, AND OCTOBER, 1866.

By N. S. DAVIS, M.D., of Chicago.

Read before the Chicago Medical Society.

According to previous report, the last three days of July were cool, clear, and pleasant. It remained cool, with a prevalence of north and north-east winds, until the morning of the 8th of August. Showers of rain fell on the 3d, and a steady rain all the night of the 6th and 7th. On the morning of the 8th, the wind had changed to the south; the air was filled with mist or aqueous vapor, and, at midday, was very warm and oppressive. Between 5 and 6 o'clock P.M., the wind suddenly changed to the north, and the atmosphere became so cool as to produce chilliness. It remained cool and clear until the 11th, when the wind changed to the south, the sky became cloudy, and in the evening there fell copious showers, with sharp lightning. The 12th was mostly clear, wind south, and atmosphere exceedingly warm and damp. These conditions continued until 11 o'clock A.M. of the 13th, when the wind again changed to the north, and the air became cool and clear. But at midnight, the wind again changed to the south, and the morning of the 14th was hot, damp, and oppressive, with showers of rain in the afternoon. From this time to the 20th, the atmosphere was still, or moved only slightly by winds from the south or south-east, very damp, and moderately warm. During the 17th and 18th, especially, the air was still and so light that smoke and mist hung close to the earth, instead of rising or of being lifted to the higher regions. Rain fell very moderately in the evening of the 18th, and during the afternoon of the 20th. During the 21st, 22d, and 23d, the wind blew from the north, and the atmosphere was decidedly cold but damp, and steady rain nearly all of the 23d. During the 24th and 25th, the wind continued north, and the air was clear, cold, and dry; so cold, indeed, that an overcoat was required for comfort during the whole day.

From the morning of the 26th to the 31st, the atmosphere was again still, damp, and moderately warm; very similar to that from the 14th to the 20th. It was filled with smoke and mist, especially during the nights. The slight winds that were felt were from the south and south-west, except during the afternoon of the 28th, when it blew from the north-east, and was cooler. Slight rains fell on the 29th and 30th, and in the evening of the 31st, copious showers fell, with thunder and lightning, during which the wind changed to the north-west and blew up a stiff breeze. Vivid flashes of lightning continued until a late hour in the night. It was the first display of the kind during the month.

From the 1st to the 25th of September, the weather was almost continuously cool and rainy, with a prevalence of north and north-east winds. The rains were so frequent and copious that the surface of the earth was kept constantly saturated with fresh fallen water. Occasionally the sun would shine out clear and warm for two or three hours in the middle of the day, but during the whole time named there were not three consecutive warm, dry days. From the 26th of September, however, until the 7th of October, the atmosphere was clear, pleasant, and moderately warm, though the prevalent winds were continued from the north, north-west, and north-east. On the 7th, the wind changed to the south, and became very light. From the 7th to the 13th, the atmosphere was filled with mist and smoke, and so still as to be scarcely moved by a breeze, either day or night. The sky was most of the time clear, and the temperature moderately warm. On the 13th, a light breeze sprung up from the north-east, the air was more bracing, and the fog or mist that had so steadily filled the lower strata of the atmosphere was dissipated in twenty-four hours. From this to the evening of the 19th, the weather was mostly clear and pleasant though warm, and very little wind from any quarter. On the 20th, the wind changed to the south-west and blew strongly, followed by clouds and some rain. The following night, there were copious showers, with thunder and lightning and strong wind from the west. The 22d was cold, nearly clear with a strong west wind, and frost at night. The 23d was cold, cloudy, with light falls of snow, sufficient to whiten the sidewalks. At this date, the evening of the 24th of October, it remains cold, cloudy, with north-east wind.

During the first seven days of August, cases of typhus fever and dysentery continued numerous, but attacks of cholera infantum and serous diarrhœa were less frequent than in the middle of July. On the 8th, there was, in my practice, a sudden increase of the latter class of affections; and a still more marked increase on the 12th, when I met with one case of cholera at 109 North Water Street, and another on Wabash Avenue, south of Twelfth Street.

On the 6th of August, a company of Mormons left one of their number, a native of Denmark, sick at the railroad depot, from whence he was taken to the County Hospital, located between 18th and 19th Streets, where he died in the evening of the same day with all the symptoms of epidemic cholera. On the 9th, three days after the Dane died, a female nurse and a male inmate of the hospital were attacked. From this time, one or more cases occurred daily among the inmates of the hospital until the 18th, when it ceased. Whole number of cases occurring in the institution, from the 6th to the 18th, was 19; of whom 11 recovered and 8 died. Nearly simultaneous with this outbreak in the County Hospital, cases of cholera occurred in almost every section of the city.

On the morning of the 14th, I was called to three cases widely separated from each other. One was at 63 Michigan Avenue, and another at 323 South Morgan Street. From this time until the 20th, new cases occurred daily in all parts of the city, although the aggregate number did not exceed an average of 20 per day. From the 20th to the 26th, there was a decided decrease in the number of cases, followed, however, by a moderate increase again from the latter date to the end of the month. The whole number of deaths from cholera during the month, as reported at the Health-Office, was 139, or a little more than 4 per day.

From the 1st to the 25th of September, the disease continued pretty nearly a uniform ratio of prevalence, although it was restricted more closely to the population of foreign birth, and in parts of the city least influenced by drainage and cleanliness. For instance, the whole number of deaths reported from this disease during the month was 166, or an average of 4 per day. Of these, 88 were natives of Germany, 40 of Ireland, and only 20 of the United States. During the last week of September, the disease declined so much that many regarded it as substantially extinct. So true was this, that the editors of the *Chicago Medical Journal* inserted the following paragraph in their issue of the 1st of October:—"In our next issue we intend to give our readers a complete sketch of the course of cholera in Chicago during the present season. The number of cases has been comparatively small, and is rapidly approaching a minimum."

The decline in the prevalence of the disease continued until about the 7th of October, when it began to increase so rapidly that from 18 cases and 7 deaths, reported on the 7th, it reached 66 cases and 17 deaths on the 11th, and 62 cases and 24 deaths on the 12th. This seemed to be the climax of its prevalence, and its decline was almost as rapid as its access. Thus, three days subsequently, namely, on the 15th, the whole number of cases was 38, with 10 deaths; and on the 17th, only 26 cases and 3 deaths. The whole number of cases reported to the Health-Officer, from the 7th to the 21st of October, was 522, of whom 135 only proved fatal. During this sudden exacerbation of the disease, it continued to manifest the same predilection for the population of foreign birth, living in undrained and uncleanly localities, as during the month of September. Thus, of the 522 cases reported, only 130 were represented to be natives of the United States.

If we compare the foregoing figures with the fact that this city contains a population of over 200,000, it will be seen that the prevalence and fatality of cholera during the past season has been far less, comparatively, than during former epidemics.

Although the prevalence of cholera in this city, during the last three months, has been very moderate in comparison with other Western cities, or with former epidemics here, yet, in all its features, it has been identically the same disease as in 1849 or 1854. The whole number of cases in proportion to the population has been very small, but the severity of such cases as have come under my observation, has been fully equal to those occurring in the former epidemics. I have not been able to trace a single new feature in relation either to the symptoms, pathology, or tendencies of the disease.

*Treatment.*—After three months of daily observation at the bedside of cholera patients, you may expect something from me in regard to the treatment of this dreaded disease. When this subject was under discussion in the Society several months since, I expressed freely my views in regard to the nature of cholera and the details of its treatment; and those views were published in the April number of the CHICAGO MEDICAL EXAMINER. Since then, I have watched, with much care, the effect of different remedies and different modes of treatment. I have seen some cases treated during the active stage with alcoholic exhilarants; others, with opiates; and still others, with salines and evacuants. A young woman, previously healthy, was attacked at breakfast-time, in the morning, with severe symptoms of cholera. A physician reached her in about two hours, and commenced, immediately, the use of brandy. He gave it in moderate, but frequently repeated, doses, until, in from three to four hours, she had taken a full pint of the brandy and some wine. At the end of that time she was in perfect collapse, pulseless, cold, and blue. No other medicine had been given. In four or five hours more she was dead. In no case have I been able to detect the slightest beneficial effect from this class of agents. Those cases, to which I have been called, in which opiates had been exclusively or chiefly relied upon during the active stage, have either had the discharges only temporarily checked, or have exhibited a persistent tendency to stupor, suppression of urine, hiccough, and ultimately death. Similar results have followed a reliance on the various cholera mixtures sold by druggists, consisting of opium, camphor, cayenne pepper, and sometimes rhubarb, in varying proportions. I saw four cases that had been treated by evacuants. The first was a man who, during the early part of the active stage, drank freely of seidlitz powders. He passed rapidly into a state of collapse and died. The second was a man who said he had taken, at the commencement of cholera symptoms, an emetic, followed by a dose of sulphate of magnesia, and still later by a dose of castor-oil. He did not go into complete collapse, but lingered three or four days, in a state of great exhaustion, and then died, the stomach appearing to have lost all power to absorb even the blandest articles of nourishment. The third and fourth cases had been treated with repeated doses of castor-oil and small doses of brandy. Both terminated fatally. From all my observations the present season, I am forced to the same conclusions in relation to the nature and treatment of the disease as in 1854, and as fully expressed in the former discussion in this Society.

From the 1st of August to the 28th of October, 205 cases of what I regarded as cholera came under my observation. Of these, 102 were in the first stage, or that of simple cholera diarrhœa, of which all but one recovered. When first seen, 77 were in second stage, or that of active vomiting, purging, cramps, etc., of which 18 died, and 59 recovered, When first seen, 26 were in the third stage, or that of complete collapse. By complete collapse, I mean a condition in which the pulse is no longer felt at the wrist; the surface is cold, corrugated, and purple or leaden color; the urine suppressed, etc. Of the 26 seen in this condition, only 2 recovered. One of the two that recovered from collapse was a boy about 7 years old. He lingered in a state of extreme feebleness for ten or twelve days, and presented well-marked ulceration in the lower half of the cornea, in both eyes. His recovery was ultimately complete. The other was a young man residing at 386 Indiana Street.

In making up the foregoing statistics of cholera cases coming under my observation, I have not included any cases of children under two years of age. The prevalence of *cholera infantum* was very severe in July, less in August, and still less in September and October. The treatment on which I have relied in the management of cholera this season, has been substantially the same as I found most efficient in the former epidemics, and, consequently, I need not repeat it here. The question, how far the prevalence of cholera this season has thrown light upon its essential causes, and its relation to certain other diseases, will be considered more fully in another communication to this Society.

---

Easily Frightened.—It seems that when the cholera began rather suddenly to increase in this city during the 8th and 9th of October, a part of the Faculty and students in Rush Medical College became decidedly alarmed; and on the morning of the 10th, at the suggestion of members of the Faculty, a large majority of the students voted to have the lectures suspended, that they might immediately leave the city. A suspension, consequently, actually took place, and the lectures were not resumed until the 22d of the same month. When it is remembered that the highest number of deaths from cholera in any one day during the present season was 24, in a population of 200,000, it must be confessed that our neighbors in Rush Medical College were rather easily frightened.

---

Poisonous Principle of Mushrooms.—Dr. Letelleir says, that the poisonous substance in mushrooms is a fixed, non-crystallizable, narcotic principle—amanitine. It is precipitated by iodine and tannin. The treatment for poisoning thence resulting is vomiting and purging, followed by a strong aqueous solution of tannin.—*British Medical Journal.*

---

Cryptogamous Origin of Intermittent Fevers.—The editors of the *Atlanta Medical Journal* state that a friend, led by the experiments of Dr. Salisbury, is investigating this subject, and will probably communicate something valuable in regard to it.

---

Obituary Record.—Died, in Boston, Sept. 15th, 1866, of cholera, after an illness of only a few hours, Augustus A. Gould, M.D., a very eminent physician and accomplished naturalist, and late President of the Massachusetts Medical Society.

MORTALITY FOR THE MONTH OF SEPTEMBER.—

CAUSES OF DEATH.

| | | | |
|---|---|---|---|
| Accidents, | 14 | Fever, Scarlet, | 12 |
| Apoplexy, | 2 | Fever, not stated, | 4 |
| Burned, | 1 | Flux, | 1 |
| Cancer, | 4 | Hydrocephelus, | 3 |
| Childbed, | 5 | Inflammation of Kidneys, | 1 |
| Cholera, | 166 | Inflammation of Brain, | 4 |
| Cholera-Morbus, | 36 | Inflammation of Bowels, | 14 |
| Cholera Infantum, | 6 | Inflammation of Lungs, | 7 |
| Colic, | 1 | Inflammation of Stomach, | 1 |
| Congestion of Brain, | 2 | Killed, | 3 |
| Consumption, | 29 | Measles, | 14 |
| Convulsions, | 20 | Marasmus, | 1 |
| Croup, | 6 | Old Age, | 13 |
| Chicken-Pox, | 1 | Phthisis Pulmonalis, | 1 |
| Disease of Brain, | 1 | Pneumonia, | 2 |
| Disease of Heart, | 11 | Scrofula, | 2 |
| Disease of Liver, | 1 | Small-Pox, | 1 |
| Disease of Kidneys, | 1 | Stillborn, | 6 |
| Delirium Tremens, | 4 | Summer Complaint, | 87 |
| Decline, | 1 | Suicide, | 3 |
| Dropsy, | 11 | Teething, | 26 |
| Diarrhœa, | 23 | Tuberculosis, | 1 |
| Diphtheria, | 9 | Whooping-Cough, | 40 |
| Dysentery, | 13 | Worms, | 1 |
| Drowned, | 7 | Wound, | 2 |
| Epilepsy, | 2 | Unknown, | 53 |
| Fever, Typhoid, | 35 | | |
| Fever, Childbed, | 2 | Total, | 739 |
| Fever, Remittent, | 16 | | |

AGES OF THE DECEASED.—Under 5 years, 329; over 5 and under 10 years, 48; over 10 and under 20, 36; over 20 and under 30, 77; over 30 and under 40, 106; over 40 and under 50, 68; over 50 and under 60, 40; over 60 and under 70, 22; over 70 and under 80, 3; over 80 and under 90, 3; unknown, 7. Total, 739.

NATIVITIES.

| | | | | | |
|---|---|---|---|---|---|
| Chicago, | 311 | Ireland, | 83 | Russia, | 1 |
| Other States, | 108 | Norway, | 25 | Switzerland, | 1 |
| Canada, | 3 | Bohemia, | 17 | Wales, | 1 |
| Denmark, | 2 | Scotland, | 4 | Sweden, | 15 |
| England, | 15 | Finland, | 2 | Unknown, | 8 |
| Germany, | 122 | Itlay, | 1 | | |
| France, | 4 | New Brunswick, | 1 | Total, | 739 |
| Holland, | 6 | Poland, | 3 | | |

---

CHOLERA HOSPITALS.—The following is the substance of a report drawn up by the Council of the Epidemiological Society, and is of unusual interest at the present time:—

I. There appears to be a general concurrence of opinion, expressed or implied, that, under certain circumstances and conditions, cholera is liable to be communicated from person to person; the liability being usually in proportion to the crowding of many persons together, the defective ventilation of apartments, and the neglect of thorough cleanliness in respect of person or abode.

In addition to the possible risk of the extension of the disease from this source, the alarming character of the symptoms, and the necessity for unremitting attendance upon the sufferers, are calculated to produce terror in the minds of spectators, and thus strongly predispose them to be attacked during an epidemic season.

For these reasons, the opinion is very generally held that it is unadvisable that cholera patients should be admitted into wards which are occupied by other sick inmates.

The experience, however, of some of the metropolitan hospitals in past epidemics, shows that, due attention being paid to sanitary arrangements, cholera patients may be received, in limited numbers, into the general wards without injurious results either in the other sick or to the ordinary attendants.

No instances have been referred to, in the evidence before the Council, in the opposite direction, *viz.:*—of the disease having spread to the other inmates of a ward in a well-regulated hospital.

II. With respect to the second query, the experience of the metropolitan physicians who have favored the Council with replies, appear to be that, with proper precautions, cholera patients may be admitted into separate wards in general hospitals or infirmaries without undue risk of the extension of the malady to the other inmates of the institution.

This opinion is shared by all the respondents who have had experience of the disease in tropical countries.

It would have been very desirable to have been informed of the results on this point, in some of the military and naval hospitals in this country and also abroad.

The precautions above referred to are these:—(*a*) Ample space to each patient; not less than 1500 or 2000 cubic feet. (*b*) Thorough ventilation of the wards at all times, both night and day. (*c*) Immediate disinfection and removal of the excreta, soiled linen, etc. (*d*) A separate staff of nurses.

III. The reply to the third query depends much on the opinion formed in respect of the two former questions. If cholera patients are not admissible into general hospitals or infirmaries under any conditions, it is obvious that some extemporized and special arrangements must be provided for the reception of the destitute when attacked.

But even when they are admitted, there are various circumstances in which it will be advisable or necessary that special hospitals should be provided, *e.g.* (*a*) When general hospitals or infirmaries are at a distance from the seat of the actual or apprehended outbreak. (*b*) When there is a want of accommodation, with due regard to the ordinary patients, or when the accommodation is unsuitable or objectionable.

In selecting the site of special hospitals, the following points require to be attended to:—(*a*) Nearness, if possible, to the chief seat or seats of the outbreak. It is important that cholera patients should not have to be carried far. There is, moreover, great risk in moving patients in, or verging to, the state of collapse (*b*) Airiness, and freedom from intrinsic or contiguous sources of atmospheric pollution. (*c*) A dry soil and raised situations are, of course, always to be preferred to a low and damp one.

Amid the crowded districts of a large town, it appears preferable that several small and suitable hospitals, or "houses of recovery," should, if possible, be established in different localities, rather than one or two large hospitals for the reception of a large number of cholera patients.

The remark that the presence of an experienced staff of medical officers in general hospitals, and the existence of more complete appliances of every sort in them than is likely to be provided in extemporized special hospitals for the treatment of cholera patients, are marked advantages in favor of the former, deserve consideration.

The general conclusions of the Council are these:—

1. That it is, on the whole, unadvisable that cholera patients be admitted into the ordinary wards of general hospitals or infirmaries.

2. That cholera patients can be safely admitted into special wards in general hospitals, due precautions being taken; and therefore, that it is desirable, as an important means of providing accommodation for the destitute when attacked, that the authorities of these institutions grant this valuable benefit to the public.

3. That it will be often necessary that special hospitals be provided in aid or in lieu of general hospitals and infirmaries.

In addition to these arrangements for the accommodation of the poor when attacked with cholera, the Council would recommend that places of refuge be provided for the temporary sojourn of some of the unattacked inmates of unwholesome dwellings and localities where the disease has appeared.—*Lancet*, July 28, 1866.

Value of Sewage.—The Reclamation Company, now actively engaged on the north side of the Thames, has already tested the value of the metropolitan sewage, and contemplates its regular and systematic utilization on a farm which the company is about to purchase.

We give the following details, as the result of its first labors, which, whether viewed in a sanitary or commercial light, we take to be of the highest importance. Early in April last, a plot of waste land at Barking Creek, devoid of surface soil, was covered with common sand, brought from Mappling, near Shoeburyness, to the extent of two feet thick, on which was sown grass seed. The surface was then well irrigated with ordinary London sewage from the northern outfall. This has been repeated once or twice. The effects of this *manufacture of green meat*—for such, indeed, it may be most justly considered—has been the production of *three crops;* one cut in June and July, at the rate of sixteen tons per acre; a second, of eight tons; and a third now growing, and almost ready for the scythe! This illustration of the value of what we have for generations cast into our rivers as waste, almost surpasses belief, and, at any rate, ought to engage the serious attention, as it is now doing, of all practical economists.—*Lancet*, Sept. 8, 1866.

